# AGE TRAJECTORIES OF LYSOPHOSPHATIDYLCHOLINES IN RELATION TO ALZHEIMER’S DISEASE: FINDINGS FROM LLFS

**DOI:** 10.1093/geroni/igae098.0029

**Published:** 2024-12-31

**Authors:** Konstantin Arbeev, Olivia Bagley, Svetlana Ukraintseva, Stephanie Cosentino, Alexander Kulminski, Eric Stallard, Anatoliy Yashin

**Affiliations:** Duke University, Durham, North Carolina, United States; Duke University, Durham, North Carolina, United States; Duke University, Durham, North Carolina, United States; Columbia University Irving Medical Center, New York, New York, United States; Duke University, Durham, North Carolina, United States; Duke University, Durham, North Carolina, United States; Duke University, Durham, North Carolina, United States

## Abstract

Lysophosphatidylcholines (LPC) are phospholipids involved in apoptosis and demyelination, among other functions. They have been linked to cancer, atherosclerosis, and neurodegeneration (with potential trade-offs) and explored as early markers of Alzheimer’s disease (AD) and accelerated aging. However, relationships between longitudinal changes in LPC levels and AD risk remain poorly understood. We applied statistical approaches for joint analyses of longitudinal and time-to-event outcomes, joint models (JM) and stochastic process models (SPM), to study longitudinal dynamics of LPC in relation to AD incidence in the Long Life Family Study (LLFS) participants. In total, 4,017 measurements of 23 LPC species in 2,858 LLFS participants from the U.S., who had 93 incident AD cases and up to two LPC measurements per individual, were analyzed. In JM applications, we found sex-specific relationship (for males only) between the dynamics of LPC species and AD incidence in the model adjusted for baseline age, education, smoking, medication use, and two genetic principal components. The strongest association was found for LPC(14:0/0:0) in males (regression coefficient: 0.739, p=2.3e-23). In SPM applications, we demonstrated that various hidden biomarkers of aging, embedded within the model’s structure and indirectly evaluated from individual LPC trajectories and data on AD incidence, significantly contribute to the associations between LPC and AD. We also observed sex differences in ‘optimal’ and ‘equilibrium’ LPC trajectories, as well as in vulnerability to LPC deviations from these trajectories, and in how these relations change with age. Our findings call for further investigation into the biological mechanisms underlying the observed associations.

